# The Cerebral Hemodynamic Response to Pain in Preterm Infants With Fetal Growth Restriction

**DOI:** 10.3389/fped.2020.00268

**Published:** 2020-05-27

**Authors:** Laura M. L. Dix, Kelsee Shepherd, Graeme R. Polglase, Suzanne L. Miller, Arvind Sehgal, Flora Y. Wong

**Affiliations:** ^1^Department of Paediatrics, The Ritchie Centre, Hudson Institute of Medical Research, Monash University, Melbourne, VIC, Australia; ^2^Monash Newborn, Monash Medical Centre, Melbourne, VIC, Australia; ^3^Neonatology, Wilhelmina Children's Hospital, Utrecht Medical Centre, Utrecht, Netherlands

**Keywords:** fetal growth restriction, noxious stimulation, cerebral oxygenation, preterm infants, IUGR, cerebral hemodynamic functional response

## Abstract

**Background:** Preterm infants undergoing intensive care often experience painful procedures such as heel lance for blood sampling. Knowledge of the cerebral hemodynamic response to painful stimuli contributes to understanding of cortical pain processing and the neurovascular network in the preterm brain. Previous research has demonstrated cerebral hemodynamic responses using near-infrared spectroscopy (NIRS) after noxious stimuli in infants appropriately grown for age (AGA). But this has not been studied in infants born small for gestational age after fetal growth restriction (FGR). FGR infants differ in brain development due to utero-placental insufficiency leading to the intrauterine growth restriction, and cerebral response to pain may be altered.

**Objectives:** We aimed to compare the cerebral hemodynamic response to painful stimuli (heel lance) in FGR and AGA infants.

**Methods:** Preterm FGR infants (*n* = 20) and AGA infants (*n* = 15) born at 28–32 weeks' gestation were studied at mean ± SD postnatal age of 11.5 ± 2.4 and 10.5 ± 2.4 days, respectively. Infants had baseline echocardiographic assessment of ductus arteriosus and stroke volume. They were monitored with NIRS for changes in tissue oxygenation index (TOI, %), and oxygenated, deoxygenated, and total hemoglobin (ΔO_2_Hb, ΔHHb, and ΔTHb) in contralateral and ipsilateral cerebral hemispheres, during a heel lance.

**Results:** At baseline, FGR infants had significantly lower TOI, higher heart rate, and lower stroke volume compared to AGA infants. Most infants in both groups showed increase in each of the NIRS parameters in the contralateral hemisphere following heel lance. However, more AGA infants (6/15) showed decreased ΔTHb compared to FGR infants (1/20) (*p* = 0.016). The magnitude of cerebral hemodynamic response and time to response did not differ between FGR and AGA infants. FGR infants showed larger ΔO_2_Hb in the contralateral compared to ipsilateral cortex (*p* = 0.05).

**Conclusion:** Preterm FGR infants have reduced stroke volume and lower cerebral oxygenation compared to AGA infants in the second to third week of life. FGR infants show similar cerebral hemodynamic responses to noxious stimuli compared to AGA infants. However, FGR infants are less likely to have a cerebral vasoconstrictive response, possibly due to cerebrovascular changes following placental insufficiency and brain sparing *in-utero*.

## Introduction

Painful procedures such as heel lances or venepunctures are common in neonatal intensive care. A median incidence of 16 painful procedures per day during the first 2 weeks of life in preterm infants has been reported (1). Pain can negatively affect brain development, by altering the pain threshold, physiological responses, and pain-related behavior (2). Little is known about the cerebral response to pain in preterm neonates and clinical pain assessment can be difficult. Several bedside scoring tools have been developed to visually assess pain-related behavior and physiological changes. However, cortical responses to pain are not always associated with visible behavioral changes in preterm infants. Individual pain-related behavioral response may also vary and not necessarily correlate with cortical nociception, reducing the value of pain assessment tools (3).

Knowledge of the cerebral hemodynamics in response to painful stimuli contributes to the understanding of cortical pain processing and development of the neurovascular network in the preterm brain. Near-infrared spectroscopy (NIRS) has been used to assess the cerebral hemodynamic response to pain in appropriately grown-for-gestational-age (AGA) preterm infants, demonstrating an increase in oxygenated hemoglobin (O_2_Hb), and /or total hemoglobin (THb) within seconds in the contralateral (CL) cerebral hemisphere in most infants (4–7). These findings indicate functional hyperemia, which is the increase in local cerebral blood flow in response to neuronal activity, via neurovascular coupling (3, 8).

To our knowledge, no study has investigated the cerebral hemodynamic response to pain in infants born after fetal growth restriction (FGR). FGR infants differ in brain development, because the suboptimal placental function leads to intrauterine growth restriction and fetal hemodynamic adaptation to ensure adequate brain perfusion (brain sparing) (9). These hemodynamic changes are associated with cerebrovascular remodeling and altered vasoreactivity (10–12). In the early postnatal period, FGR infants have higher levels of cerebral oxygenation compared to AGA infants, likely due to higher cerebral perfusion secondary to brain sparing (13). However, this increased cerebral perfusion does not confer neurodevelopmental advantage. FGR infants demonstrate a significantly larger reduction in cerebral oxygenation in the presence of patent ductus arteriosus (PDA), and they are at increased risks for impaired neurodevelopmental outcome (14, 15).

As the cerebral hemodynamics in FGR infants differs from their AGA peers (16), FGR infants might also differ in their cerebral response to pain. Studying the cerebral hemodynamic functional response in FGR infants may provide insight on the influence of pain on neurovascular coupling, brain plasticity, and their cognitive or behavioral outcomes. The aim of this study was to assess the cerebral hemodynamic response to a painful stimulus elicited by heel lance in FGR infants compared to AGA infants, using NIRS. We hypothesized that FGR infants would show reduced cerebral hemodynamic responses compared to AGA infants, due to compromised cerebral vasoreactivity (17).

## Materials and Methods

### Patients

Preterm FGR and AGA infants born at 28–32 weeks of gestation age (GA) at Monash Newborn in Melbourne were included for study on or after postnatal day 7. FGR was identified based on birth weight under the 10th percentile (Growth chart, Pfizer Australia Pty Lt), and compromised fetal growth and poor placental function on prenatal scans. Exclusion criteria included evidence of intrauterine infections (e.g., TORCH infections), chromosomal abnormalities, major congenital abnormalities, or major brain pathologies such as Grade 3–4 intraventricular hemorrhage and periventricular leukomalacia. AGA infants of similar GA and postnatal age to FGR infants were recruited for comparison.

Baseline echocardiographic evaluations were performed within 24 h prior to the studies, by a single operator (AS) using the Vivid E95 cardiovascular ultrasound system (GE Medical Systems, Milwaukee, WI) and a 12 MHz phased array transducer probe that allowed image acquisition at a rate of 120–180 frames per second. PDA was assessed using 2D and color Doppler form the high parasternal ductal view. Only infants with a closed PDA on echocardiography were included to avoid the confounder of shunt effects on cerebral perfusion. Stroke volume was assessed on echocardiography from the aortic trace using pulse wave Doppler method. Long axis left ventricular outflow tract view was obtained from the apical five chamber view and the line of insonation was aligned with the flow, sample placed at tips of aortic valve leaflets. It was indexed to weight by using the formula: [(3.14 × cross sectional area × VTI)/birthweight, ml/kg]. Stroke volume was multiplied with heart rate reading obtained from the left ventricular outflow Doppler, to calculate cardiac output. Data were analyzed offline on EchoPacTM (Horten, Norway).

All infants were clinically stable at the time of study, none were being treated with inotropic medications, suffered from meningitis, or overwhelming sepsis. Perinatal demographics were retrieved from hospital records.

### Protocol

All infants were studied in the supine position with their head in the midline position, in a quiet and awake state. Arterial oxygen saturation (SaO_2_) was measured by an oximeter probe (Masimo, Australia) on the right upper limb. SaO_2_ was maintained at 90–95% for infants who required inspired oxygen, according to clinical protocol. Heart rate (HR) was recorded from electrocardiogram (CovidienTM, USA). Cerebral oxygenation was continuously measured with a two-channel NIRS (NIRO-200NX, Hamamatsu photonics K.K., Hamamatsu City, Japan) at 5 Hz, demonstrating changes in concentrations of oxygenated, deoxygenated and total hemoglobin (ΔO_2_Hb, ΔHHb, ΔTHb = ΔO_2_Hb + ΔHHb, μM.cm), and the tissue oxygenation index (TOI, %) which represents the ratio between O_2_Hb and total hemoglobin (18). The emitter and detector were placed 4 cm apart in the temporo-parietal region on each of the contralateral (CL) and ipsilateral (IL) sides with regards to the heel lance, at the F3-CP3 and F4-CP4 positions (according to the international EEG 10–20 system) with the midpoints centered over C3 and C4, respectively. The placement provides access to the representation area of the heel in the primary somatosensory cortex (4).

The noxious stimulus was a heel lance (Quikheel Preemie Lancet, BD Microtainer, NJ, USA) for routine clinical blood test, performed by experienced neonatal research nurses. Infants were swaddled prior to the heel lance as a comforting measure. None of the infants received non-nutritive sucking, oral sucrose, analgesia, or sedation at time of study. All physiological parameters were simultaneously recorded on a PowerLab system (ADInstruments, Castle Hill, Australia) at a sampling rate of 400 Hz, and analyzed as changes from the individual baselines of 20 s before the heel lance. After the heel lance, the foot was not squeezed for 30 s to ensure that the evoked response occurred only as a result of the skin breaking procedure.

### Data Analyses

Physiological signals were averaged as consecutive bins of 1-s duration (LabChart 7, ADInstruments, Australia). Changes from baseline in all signals following the heel lance were collected for at least 20 s. The maximum change and latency in cerebral NIRS measurements were identified, and the response was considered significant if a change of >2 standard deviations (SD) from the individual baseline occurred.

### Statistical Analysis

Statistical analysis was performed with SPSS 22 (IBM SPSS Statistics for Windows, V22.0, NY, USA). Clinical characteristics were analyzed with an unpaired Student *t*-test or chi-square test. Baseline TOI and stroke volume, and vital parameters of HR and SaO_2_ were compared between FGR and AGA infants using the unpaired Student *t*-test, or the Mann-Whitney test for non-parametric data. The cerebral hemodynamic response to heel lance was categorized as “increase” or “decrease,” or no-response (change <2SD compared to baseline). The chi-square test was used to compare, between FGA and AGA infants, the proportions of infants in each category of cerebral hemodynamic response, for each of the cerebral oxygenation parameters including ΔO_2_Hb, ΔHHb, ΔTHb, and ΔTOI (for CL or IL cortex). The maximum changes in the cerebral oxygenation parameters as well as time to the maximum changes were compared between FGR and AGA infants using the unpaired Student *t*-test, or the Mann-Whitney test for non-parametric data. Within each birthweight group, the paired *t*-test or Wilcoxon signed-rank test was used to compare between the CL and the IL cerebral responses, and to compare HR and SaO_2_ before and after the heel lance. Values are expressed as mean ± SD. Statistical significance was set to *p* < 0.05.

## Results

Twenty preterm FGR infants and 15 AGA controls were studied between days 7 and 17 of postnatal age. All FGR infants had estimated fetal weight below the 10th percentile for gestational age and evidence of FGR with slowing fetal growth velocity on serial prenatal scans (19). Seventeen FGR infants also had abnormal fetal multi-vessel integrated Doppler analysis (umbilical artery, middle cerebral artery, and ductus venosus), and were delivered preterm due to worsening Doppler measurements and/or abnormal cardiotocography (CTG). The other 3 FGR infants were delivered preterm for severe maternal pre-eclampsia or abnormal CTG.

Clinical characteristics of the FGR and AGA infants are shown in Table 1. As per study design, FGR infants had significantly lower birthweight than the AGA group and a lower body weight at the time of study. There were no differences between the 2 groups in GA at birth, sex, or postnatal age at time of study. More FGR infants (17/20) had respiratory distress syndrome than AGA infants (8/15). One FGR infant only had unilateral NIRS monitoring on the CL side with regards to the heel lance. This infant was included in the analysis except when comparing between the CL and IL cortical responses.

**Table 1:** Clinical characteristics.

	**FGR**	**AGA**	***p*-value**
*N*	20	15	
GA at birth, weeks	30.7 ± 1.2	30.8 ± 1.2	ns
Sex, male/female	10/10	8/7	ns
Birthweight, grams	1,018 ± 225	1,450 ± 255	<0.001
Apgar 5 min, median [range]	9 (6-9)	9 (7-9)	ns
Maternal age years	32 ± 7	32 ± 7	ns
Respiratory distress syndrome, N	17	8	0.04
**Age at Study**			
- Postnatal age, days	11.5 ± 2.4	10.5 ± 2.4	ns
- Postmenstrual or corrected GA, weeks	32.3 ± 1.23	32.3 ± 1.20	ns
Weight at study, grams	1,076 ± 297	1,481 ± 231	<0.001
Respiratory support during study, *N*			ns
- None	12	10	
- High flow	2	3	
- Continuous positive airway pressure	6	2	

*Values are in mean ± SD unless otherwise stated, GA, gestational age; ns, non-significant*.

### Cerebral Parameters in FGR vs. AGA Infant

Baseline TOI (mean ± SD) was lower in FGR compared to AGA infants (66.5 ± 4.9 vs. 70.7 ± 6.3%, *p* < 0.01), with no significant difference between their SaO_2_ levels (97.1 ± 3.3 vs. 98.0 ± 1.4%, respectively). Cerebral fractional oxygen extraction, calculated as (SaO_2_- TOI)/ SaO_2_, was higher in FGR compared to AGA infants (0.32 ± 0.06 vs. 0.28 ± 0.07, *p* = 0.026).

**Figure 1:**
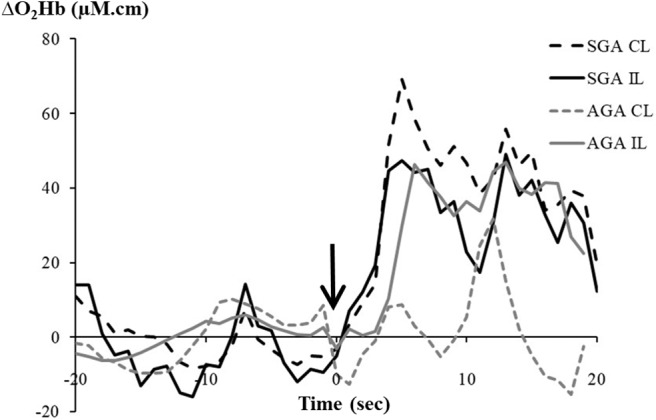
Changes in oxygenated hemoglobin (ΔO_2_Hb, μM.cm) recorded from the contralateral (CL) and ipsilateral (IL) hemispheres following a heel lance in a fetal growth-restricted (FGR) infant and an appropriate-for-gestational-age (AGA) infant. The arrow indicates timing of the heel lance.

Figure 1 shows examples of the bilateral NIRS measurement of ΔO_2_Hb in response to heel lance, in a FGR and AGA infant, respectively.

#### Contralateral Cortical Response to Heel Lance

For the CL cortex to side of the heel lance, maximum changes in cerebral NIRS parameters from the baseline following the heel lance are shown in Table 2. Overall, the majority of both FGR and AGA infants showed increased ΔO_2_Hb (FGR 11/20, AGA 10/15), ΔHHb (FGR 10/20, AGA 9/15), and ΔTHb (FGR 13/20, AGA 8/15). The proportions of infants with an increase in each of the CL cerebral parameters were similar between the FGR and AGA groups.

**Table 2:** Changes in cerebral oxygenation parameters in the contralateral cerebral cortex.

**Contralateral cortical response pattern**	**All**	**Increase**	**Decrease**	**No-response**
**FGR**, ***N*** **= 20**				
- ΔO_2_Hb (μM.cm) (*N*) - Time to max change (sec)	27.49 ± 33.37 4.85 ± 2.19	47.38 ± 16.09 (11)	−31.76 ± 42.92 (2)	13.16 ± 25.51 (7)
- ΔHHb (μM.cm) (*N*) - Time to max change (sec)	17.70 ± 20.55 7.64 ± 4.54	31.13 ± 18.03 (10)	−23.58 (1)	7.36 ± 8.83 (9)
- ΔTHb (μM.cm) (*N*) - Time to max change (sec)	42.58 ± 45.76 5.36 ± 2.44	66.36 ± 31.91 (13)	−71.52 (1^*^)	10.08 ± 13.17 (6)
- ΔTOI % (*N*) - Time to max change (sec)	0.35 ± 2.54^a^ 5.61 ± 2.47	2.08 ± 0.16 (8)	−3.05 ± 1.77 (5)	0.81 ± 1.69 (7)
**AGA**, ***N*** **= 15**				
- ΔO_2_Hb (μM.cm) (*N*) - Time to max change (sec)	8.74 ± 44.14 5.69 ± 3.38	31.94 ± 23.11 (10)	−64.89 ± 19.28 (3)	3.20 ± 4.58 (2)
- ΔHHb (μM.cm) (*N*) - Time to max change (sec)	15.99 ± 37.96 5.54 ± 3.28	37.76 ± 29.30 (9)	−27.29 ± 20.65 (4)	4.57 ± 1.39 (2)
- ΔTHb (μM.cm) (*N*) - Time to max change (sec)	21.65 ± 44.99 5.50 ± 3.46	51.49 ± 34.78 (8)	−14.55 ± 29.73 (6)	0.04 (1)
- ΔTOI % (*N*) - Time to max change (sec)	−2.71 ± 9.3^a^ 6.31 ± 3.07	2.35 ± 1.74 (6)	−7.85 ± 11.83 (7)	0.10 ± 1.80 (2)

*Values are changes relative to baseline and in mean ± SD (N, number of patients). ΔHHb, change in deoxygenated hemoglobin; ΔO_2_Hb, change in oxygenated hemoglobin; ΔTHb, change in total hemoglobin; TOI, Tissue oxygenation index*.

a *data not-normally distributed*.

* *p < 0.05 compared to AGA group*.

No significant difference was found between the two groups in the maximum changes in the CL cerebral ΔO_2_Hb, ΔHHb, ΔTHb, and ΔTOI. When focusing on only infants with increased CL ΔO_2_Hb, a trend was seen in the ΔO_2_Hb response being higher in FGR compared to AGA infants, but failed to reach statistical significance (47.38 ± 16.09 vs. 31.94 ± 23.11 μM.cm, *p* = 0.09).

ΔTHb is an important measure of cerebral blood volume and indicates cerebral vasoreactivity (20, 21), where a reduced ΔTHb is indicative of cerebral vasoconstriction in response to the heel lance. The proportion of infants who showed decreased CL ΔTHb was significantly lower in the FGR (1/20) group compared to the AGA (6/15) (*p* = 0.016, Table 2). Moreover, the proportion of infants with no-response in CL ΔTHb was higher in FGR (6/20) compared to AGA (1/15) group, but did not reach statistical significance (*p* = 0.098, Table 2).

#### Ipsilateral Cortical Response to Heel Lance

In contrast to the CL cortex, no differences were found in the IL cortex between the FGR and AGA infants in the maximum changes, or in the proportions of infants with an increase/decrease/no response in each of the cerebral parameters (data not shown).

#### Time to Maximum Changes in Cerebral Parameters

No differences were found between FGR and AGA infants (infants with no-response were excluded) in the time to reach maximum response in all cerebral parameters, in both the CL and IL cortices (Table 2).

### Comparison of Cerebral Parameters Between IL and CL Cortices

In FGR infants, change in ΔO_2_Hb after the heel lance was larger in the CL than the IL cortex (*p* = 0.05, Table 3). Their changes in ΔHHb and ΔTHb were also larger in the CL cortex compared to the IL cortex, but the differences were not statistically significant (*p* = 0.08 for both, Table 3). In AGA infants, none of the cerebral parameters were different between the IL and CL cortices (Table 3).

**Table 3:** Changes in cerebral oxygenation parameters in the ipsilateral vs. contralateral cerebral cortex.

	**Ipsilateral**	**Contralateral**	***p*-value**
**FGR**, ***N*** **= 19**			
- ΔO_2_Hb (μM.cm)	3.71 ± 47.97	28.62 ± 33.89	0.05
- ΔHHb (μM.cm)	8.60 ± 18.65	19.87 ± 18.60	0.08
- ΔTHb (μM.cm)	14.97 ± 55.58	44.54 ± 46.15	0.08
- ΔTOI %	0.00 ± 3.28	0.35 ± 2.54	0.74^a^
**AGA**, ***N*** **= 15**			
- ΔO_2_Hb (μM.cm)	28.90 ± 61.87	8.74 ± 44.14	0.18
- ΔHHb (μM.cm)	16.29 ± 38.98	15.99 ± 37.96	0.99
- ΔTHb (μM.cm)	38.89 ± 74.70	21.65 ± 44.99	0.31^a^
- ΔTOI %	−1.08 ± 4.75	−2.71 ± 9.3	0.65^a^

*Values are changes relative to baseline and in mean ± SD. ΔHHb, change in deoxygenated hemoglobin; ΔO_2_Hb, change in oxygenated hemoglobin; ΔTHb, change in total hemoglobin; TOI, Tissue oxygenation index*.

a *data not-normally distributed, non-parametric testing was used*.

When cerebral parameters were analyzed respectively for left- and right-sided heel lance in each group, no significant differences were found between the CL and IL sides, in both FGR and AGA infants (data not shown).

### Echocardiographic and Vital Parameters

Stroke volume (mean ± SD) was lower in FGR compared to AGA infants (1.41 ± 0.02 vs. 1.56 ± 0.02 ml/kg, *p* < 0.001). HR from the echocardiographic recording was higher in FGR compared to AGA infants (155.1 ± 5.7 vs. 147.3 ± 4.7 bpm, *p* < 0.001). The resulting cardiac output trended lower in the FGR cohort (218.5 ± 18.2 vs. 229.75 ± 14.6 ml/kg/min, *p* = 0.057).

During the study, HR was again higher in FGR infants compared to the AGA infants, both at baseline just before the heel lance (*p* < 0.001) and following heel lance (*p* < 0.05 (Table 4). HR increased after the heel lance in AGA infants (*p* = 0.01, Table 4), but not in the FGR infants. SaO_2_ remained unchanged in both FGR and AGA infants before and after the heel lance, and did not differ between the groups.

**Table 4:** Changes in vital parameters.

	**Before heel lance**	**After heel lance**	***p*-value^b^**
**FGR**, ***N*** **= 19**			
- SaO_2_ (%)	97.1 ± 3.4	97.5 ± 2.6	0.47^a^
- HR (BPM)	164.8 ± 12.7^**^	168.4 ± 10.8^*^	0.23
**AGA**, ***N*** **= 15**			
- SaO_2_ (%)	98.0 ± 1.4	97.3 ± 2.0	0.08^a^
- HR (BPM)	143.5 ± 15.6	156.0 ± 20.5	0.01

*BPM, beats per minute, HR, heart rate, SaO_2_, arterial oxygen saturation*.

a *data not normally distributed, non-parametric testing was used*.

b *p-value for comparison between before and after heel lance*.

* p < 0.05 and

** *p < 0.001 for comparison with AGA infants*.

## Discussion

### Summary

This study is the first to examine the cerebral hemodynamic response to a noxious stimulus in FGR infants. At baseline, FGR infants had lower TOI, lower stroke volume and higher HR than the AGA infants. After the heel lance, the majority of both FGR and AGA infants showed increased ΔO2Hb, ΔHHb, and ΔTHb in the CL cortex, with similar magnitudes of changes between the two groups. However, FGR infants were less likely to show cerebral vasoconstriction following the heel lance, as evidenced by 1/20 FGR and 6/15 AGA infants showing decreased ΔTHb. Furthermore, HR was higher following heel lance in AGA infants suggestive of a systemic response, but this did not occur in the FGR infants. Taken together, these results suggest that the cerebral and systemic hemodynamic response to noxious stimuli might be blunted in FGR infants. In addition, AGA infants showed similar ΔO_2_Hb between their CL and IL cortices after heel lance, while FGR infants showed relatively larger increase in ΔO_2_Hb in the CL compared to the IL cortex, suggesting altered neurovascular development in the FGR brain. Overall, our data adds to the growing body of evidence that FGR infants show different vascular properties compared to AGA infants (9, 17).

### FGR Infants Have Lower Baseline Cerebral Oxygenation and Stroke Volume

Prenatal Doppler sonography has demonstrated lower cerebral vascular resistance in FGR infants, suggesting fetal cerebral vasodilation as a result of brain sparing. There is evidence to indicate that fetal cerebral vasodilation persist postnatally, with increased cerebral blood flow (22) and lower resistance of the cerebral arteries up to 4 days after birth (23, 24). FGR infants also have higher regional cerebral oxygen saturation upto 3 days of postnatal age (13, 21). These cerebral hemodynamic parameters show normalization after a few days, indicating that the cerebral circulatory changes are transitory (21–23). However, to-date there is little postnatal research to follow up the cerebral hemodynamics in FGR infants beyond the very early postnatal period. The lower baseline TOI in our FGR infants in the 2nd to 3rd weeks of life may indicate reduced cerebral blood flow due to their lower stroke volume, secondary to the subclinical alterations in systolic and diastolic function reported in FGR fetuses (25) and infants (26, 27). In addition, the lower stroke volume may be due to a higher systemic vascular resistance, as sympathovagal imbalance with a relative increase in sympathetic activity has been reported in growth-restricted infants up to 3 months of age, which also contributes to the higher HR as we and others have found (26, 28). In spite of a higher HR in the FGR population, the cardiac output still trended lower. On the other hand, the higher cerebral oxygen extraction in the FGR infants indicates a higher cerebral oxygen consumption relative to supply. It is possible that the FGR brain has increased metabolic demand due to neuroinflammation resulted from the chronic hypoxia *in-utero* (9).

### FGR Infants Are Less Likely to Show Cerebral Vasoconstrictive Response After Noxious Stimuli

Increased ΔTHb following the heel lance indicates an increase in the cerebral blood volume, representing functional hyperemia in response to the neuronal stimulation. However, previous studies in newborn infants show that the ΔTHb may increase, decrease or remain unchanged after a heel lance, suggesting that functional hyperemia may not always occur in the immature brain (4, 5, 7). Reduced ΔTHb with cerebral vasoconstriction has also been reported in neonatal rats after hindpaw electrical stimulation (29). Forty percent of our AGA infants showed decreased ΔTHb in response to the heel lance, which suggests cerebral vasoconstriction. Significantly less FGR infants showed reduced ΔTHb compared to the AGA group. On the other hand, only 1 AGA infant showed no change in ΔTHb after the heel lance, which might reflect the lack of cerebrovascular response to the noxious stimulus. The proportion of infants with no change in THb was higher in the FGR group (6/20), though not statistically significant. These results suggest that FGR infants were less likely to mount a cerebrovascular response, and also less likely to have cerebral vasoconstriction following a noxious stimulus. Chronic cerebral vasodilation from brain sparing due to FGR could contribute to this altered cerebrovascular response. In addition, HR increased significantly after the heel lance in the AGA infants, but not in the FGR infants. This might also reflect an altered and blunted autonomic cardiovascular response in the FGR infants.

### FGR Infants Showed Relatively Larger Hemodynamic Changes in the CL Than IL Cortex

Although the magnitudes of changes in cerebral hemodynamics after the heel lance were similar between the FGR and AGA infants, the relative changes between CL and IL cortices were different between the 2 groups. Several studies in preterm AGA infants (4–6) showed increases in O_2_Hb and THb in the CL cortex in response to painful stimuli, while IL cortical changes were variable and smaller in magnitude. Our FGR infants showed larger changes in the CL than IL cortex, consistent with previous studies in AGA infants. In contrast, the AGA infants in our study had similar cerebral hemodynamic changes in the CL and IL hemispheres. This could be due to our smaller GA range compared to previous studies. Bartocci et al. showed that the magnitude of O_2_Hb increase was negatively correlated with GA, but more so in the left hemisphere (6). Moreover, the O_2_Hb response was greater in the CL than the IL side only with right-sided noxious stimuli, indicating the dominance of the hemodynamic response in the left hemisphere. Hence, their results could be affected by the infants at younger GA with right-sided noxious stimuli. We therefore analyzed the infants with left or right-sided heel lance separately, but found no relative difference between CL and IL responses.

The bilateral cerebral hemodynamic response following somatosensory stimuli, as seen in our AGA infants, has been described by studies in term neonates using magnetic resonance imaging (30, 31). Development toward co-activation of the IL somatosensory cortex was seen with increasing postmenstrual age (30, 32), and may be due to astrocyte development and maturation of the neurovascular network to influence blood flow over a larger area (33). As such, the larger CL than IL hemodynamic changes in our FGR infants might indicate a relatively less developed brain compared to their AGA peers (34). Delayed brain maturation has been shown in FGR infants, with decreased brain volume, discordant gyrification and decreased myelination (9, 35, 36). Such chronic cerebral changes in FGR infants may also alter the neurovascular coupling and the cerebral hemodynamic response to pain.

### Latency of the Cerebral Hemodynamic Response

In term newborns, increased O_2_Hb and THb were observed at ~3–4 s following heel lance (4). In preterm infants born between 24 and 37 weeks' GA, averaged latency of the THb response was at ~8 s, and this latency decreased with increasing age (5). Consistent with these studies, our AGA and FGR preterm infants of 28–32 weeks' GA showed a latency of ~5–7 s. A study using venepuncture in preterm infants of 28–36 weeks' GA found an increase in cerebral O_2_Hb starting at 2 s with the latency at ~40 s after the needle insertion (6). This type of noxious stimulus is different from the heel lance we used and lasts much longer, which might explain the difference in results.

### Limitations

Our study has several limitations, including the small study population which restricts data interpretation. The cerebral hemodynamic response in the FGR infants implies cortical processing of pain. Notably, cerebral hemodynamics are coupled to the neuronal firing activity in synaptic terminals, regardless of whether excitatory, or inhibitory neurotransmitters are released. Therefore, any correlation with pain intensity or other perceptual qualities of acute pain is speculative and cannot be based on these data. Cerebral hemodynamic responses can be affected by other factors, such as hemoglobin levels or arterial carbon dioxide tension. However, both groups were clinically stable and either self-ventilating or requiring low level of respiratory support, making these influences negligible. Most infants were asleep at the start of the study. Sleep states can affect the preterm cerebral oxygenation (37). Crying in response to the heel lance may also exert physiological effects on cerebral perfusion, blood pressure and intrathoracic pressures with consecutive influence on venous return or cardiac output. We did not record the sleep state or perform behavioral pain assessment, and therefore could not assess the effects of sleep state or pain-related behavior on the cerebral hemodynamic response. Our study population was limited to clinically stable preterm FGR. Whether the same cerebral hemodynamic response occurs in the sicker and more unstable FGR infants including those requiring higher levels of ventilatory support and/or inotropic medications remains to be investigated. To-date there is minimal information on the relationship between cerebral hemodynamics after birth and neurodevelopmental outcome in FGR infants. Future research in a larger population of infants would be useful to investigate the cerebral and systemic response to pain in FGR infants, incorporating both physiological, and behavioral assessments, in relation to the long-term outcomes.

## Conclusion

Our study provides new physiological insights in the cerebral hemodynamics in FGR infants, which add to the understanding of their brain development, cerebrovascular remodeling, and vasoreactivity. The FGR infants had lower cerebral oxygenation compared to AGA infants by the 2nd week of life, possibly related to subclinical cardiovascular dysfunction. Following a noxious stimulus, FGR showed similar changes in cerebral hemodynamic to AGA infants, suggesting similar cerebral processing of pain. However, FGR infants are less likely to have a cerebral vasoconstrictive response, possibly due to the chronic cerebral vasodilation *in-utero*. FGR infants also showed relatively larger hemodynamic changes in the CL compared to IL cortex, suggesting altered neurovascular development.

## Data Availability Statement

The datasets generated for this study are available on request to the corresponding author.

## Ethics Statement

The studies involving human participants were reviewed and approved by Monash Health Human Research Ethics Committee. Written informed consent to participate in this study was provided by the participants' legal guardian/next of kin.

## Author Contributions

LD: conducted the study, collected the data, analyzed the data, wrote the first draft of the manuscript, and edited revisions. KS: conducted the study, collected the data, analyzed the data, and reviewed the manuscript. GP and SM: initiated the study and reviewed the manuscript. AS: designed the study, performed echocardiography on all infants, analyzed the data, and reviewed the manuscript. FW: initiated the study, designed the study, analyzed the data, edited revisions, and reviewed the manuscript.

## Conflict of Interest

The authors declare that the research was conducted in the absence of any commercial or financial relationships that could be construed as a potential conflict of interest.

## Abbreviations

AGA: Appropriately grown for gestational age
CL: Contralateral
FGR: Fetal growth restriction
GA: Gestational age
HHb: Deoxygenated hemoglobin
IL: Ipsilateral
O_2_Hb: Oxygenated hemoglobin
THb: Total hemoglobin
TOI: Tissue oxygenation index.
